# Connecting the Neurobiology of Developmental Brain Injury: Neuronal Arborisation as a Regulator of Dysfunction and Potential Therapeutic Target

**DOI:** 10.3390/ijms22158220

**Published:** 2021-07-30

**Authors:** Ane Goikolea-Vives, Helen B. Stolp

**Affiliations:** Department of Comparative Biomedical Sciences, Royal Veterinary College, London NW1 0TU, UK; agoikoleavive18@rvc.ac.uk

**Keywords:** dendritic arborisation, dendritic spine, synapse formation, neurodevelopmental disorder, perinatal brain injury

## Abstract

Neurodevelopmental disorders can derive from a complex combination of genetic variation and environmental pressures on key developmental processes. Despite this complex aetiology, and the equally complex array of syndromes and conditions diagnosed under the heading of neurodevelopmental disorder, there are parallels in the neuropathology of these conditions that suggest overlapping mechanisms of cellular injury and dysfunction. Neuronal arborisation is a process of dendrite and axon extension that is essential for the connectivity between neurons that underlies normal brain function. Disrupted arborisation and synapse formation are commonly reported in neurodevelopmental disorders. Here, we summarise the evidence for disrupted neuronal arborisation in these conditions, focusing primarily on the cortex and hippocampus. In addition, we explore the developmentally specific mechanisms by which neuronal arborisation is regulated. Finally, we discuss key regulators of neuronal arborisation that could link to neurodevelopmental disease and the potential for pharmacological modification of arborisation and the formation of synaptic connections that may provide therapeutic benefit in the future.

## 1. Introduction

Dendritic arbours, together with dendritic spines in spiny neurons, are fundamental in regulating both the information received by a neuron and the way that this information is processed and acted upon. As a result, changes in dendritic arborisation, or the formation of dendritic spines, have a dramatic effect on brain function. This is evidenced by a substantial body of research correlating alterations in dendrites and dendritic spines with the severity of cognitive and behavioural symptoms of neurodevelopmental, neuropsychiatric, and neurodegenerative disorders.

Dendrite formation is a relatively late and extended developmental event, after a prolonged period of proliferation, that follows a broadly stereotypic pattern for all neurons. It is driven by a combination of intrinsic genetically regulated processes, particularly important during early phases of neurite extension, that are then dynamically influenced by a multitude of extrinsic cues, including activity-dependent regulation [1,2,3]. For the identification of potential therapies to correct disrupted arborisation and connectivity in neurodevelopmental disorders, it is necessary to understand the consequences of genetic and environmental events on dendritic arborisation, the time-dependence of these disruptions, and the capacity for structural or functional compensation as part of normal development. While progress is being made in our understanding of many of these areas, there is still a lack of overview and integration of information necessary to make the required progress in therapeutic discovery. To facilitate this progress, we will review the links between neurodevelopmental disorders and disrupted dendritic development, considering the potential consequences of disruption for the functioning of neural networks. The mechanisms underlying alterations in dendritic and synaptic density in neurodevelopmental disorders will be explored, particularly focusing on those mechanisms that show promise for therapeutic intervention.

## 2. Disrupted Neuronal Arborisation in Neurodevelopmental Disorders

**Figure 1:**
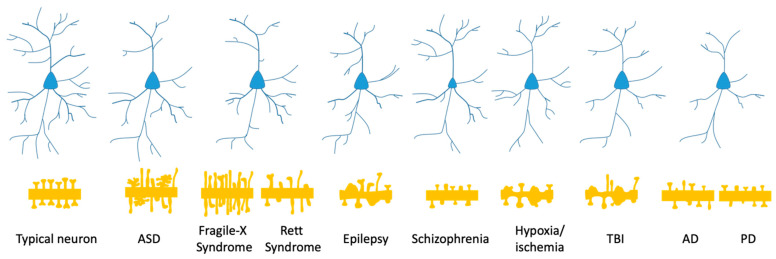
Schematic diagram summarising disease associated alterations in dendritic arborisation and spine formation. A neurotypical neuron elaborates complex branching and long and numerous dendrites. It also develops relatively stable and mature spines. Individuals with ASD often show reduced dendritic branching complexity and increased presence of immature/thorny spines and increased spine density. Neurons from fragile X and Rett syndrome patients have been found to display shorter and less abundant dendrites and abnormally long and thin spines of increased density. In patients with epilepsy, neurons have been observed to form shorter and less branched arbours that often display varicosities and distorted spines, and spine density is also decreased. Neurons from schizophrenic individuals display decreased spine size and abnormal spine necks, smaller somas, and a reduced number of dendrites. In subjects with hypoxia/ischemia or TBI, neurons elaborated less and shorter dendrites, with varicosities and constrictions, and showed reduced spine density and presence of abnormal spines. Decreased dendritic length and branching as well as spine loss has been observed in neurons from patients with AD and PD. AD—Alzheimer’s disease, ASD—autism spectrum disorder, PD—Parkinson’s disease, TBI—traumatic brain injury.

Neuronal morphology is a major determinant of neuronal connectivity and normal brain function [4,5]. The dendritic branching pattern, as well as dendritic and spine density, size, and morphology, determines the efficacy of the synaptic input transmission, integration, and processing [5,6]. Many neurodevelopmental pathologies exhibit dendritic and spine abnormalities, summarised in Figure 1 [7,8,9,10]. For instance, brain post-mortem studies from autistic patients reported reduced dendritic branching complexity in the hippocampal CA1 and CA4 regions [11], a reduction in the number of dendrites in the dorsolateral prefrontal cortex [12], and increased spine densities in cortical pyramidal neurons [13]. Dendritic abnormalities are a core feature of syndromes such as Down syndrome, Rett syndrome, fragile X syndrome, and phenylketonuria; patients displayed a decreased number and length of dendritic arbours as well as abnormal morphology and number of dendritic spines in the cerebral cortex (reviewed in [14]). Disorders such as epilepsy and traumatic brain injuries (TBI), in which excitotoxity is involved, have also been associated with aberrant dendritic spine structure and distribution [15]. Post-mortem reports from patients with epilepsy showed decreased dendritic branching complexity, fewer branches, as well as decreased spine density and dendritic swelling in layer III cortical pyramidal neurons [16]. Dendritic varicosities and loss of dendritic spines have been observed in the hippocampus of these patients [17]. Perinatal hypoxic/ischemic brain injury can result in long-term neurologic defects or death of the new-born (reviewed in [18]). Animal studies have shown that hypoxic-ischemic events lead to the loss of dendritic spines, appearance of dendritic varicosities, reduced dendritic length, and dendritic branching in rat cortical pyramidal neurons [19,20] and sheep cortical and subcortical neurons [21,22,23,24].

## 3. Neuronal Arborisation and Synapse Formation as Part of Cortical Circuit Formation

The earliest cortical circuits in humans are formed in the preplate by gestational week 5 [25,26,27,28]. Neurons within the preplate create primitive and temporary synaptic connections with adjacent cells acting like provisional targets until migrating neurons arrive to form more stable connections. These preplate neurons are also the first neurons to project outside the cerebral cortex. As the cortical laminae develops, neurons generate short- and long-distance connections to create local and globally interlinked neural networks. Developmental processes following migration and differentiation initially result in excessive neuronal arborisation and synaptic connectivity. These require refinement, first by spontaneous activity and later by extrinsic stimuli-dependent activity, in order to form and establish mature neural circuits [29]. Spontaneous neuronal activity is necessary for the initial development of connectivity, and it reaches the cerebral cortex through the thalamocortical pathway, even before the radial migration of cortical neurons has been completed [30]. Seminal studies conducted in the visual system of prenatal cats demonstrated that blockage of spontaneous firing of action potentials before eye opening impaired normal axon terminal branching of retinal ganglion cells [31] and of thalamocortical pathway neurons, which led to the aberrant formation of ocular dominance columns in the primary visual cortex [32]. In the developing mouse somatosensory cortex, before any sensory stimuli can be received, the absence of spontaneous activity that originates from the thalamus results in cortical hyperexcitable circuits and aberrant development of functional columnar structures [33].

As the brain matures and begins to receive sensory input, the number, type and strength of synapses varies as a result of neuronal activity. This neuronal activity not only enables the addition of unique information into neuronal patterns, but also promotes circuit refinement and is essential in the development of mature circuitry. After birth, dendritic morphogenesis is particularly susceptible to activity-dependent inputs, and it is crucial to determine neuronal dendritic structure and the type of connections to establish [34]. Additionally, dendritic branches can remodel in response to damage caused by injury or disease. The capacity to reshape and adapt to change is termed plasticity, and it has been shown to be present throughout adult life [35,36].

During the first 18 months of life, the rate of dendritic morphogenesis and synaptogenesis increases, and developmental processes such as experience-dependent synapse remodelling and pruning approach a critical period in which incorrect timing and rate has been proposed to lead to the development of several neurodevelopmental disorders [26,37,38]. Dendritic and synaptic pruning is driven by an interplay of neurons, microglia and astrocytes [39]. Pruning occurs in two phases: directly after birth—early childhood—to ensure the correct formation of sensory circuits; and during the transition from childhood, adolescence, and adulthood to remodel circuits involved in higher cognitive functions including self-regulation [39,40]. Abnormal pruning leads to aberrant dendritic arborisation and synaptic function.

Interestingly, it appears that dendrite maturation and expression of behavioural symptoms of some neurodevelopmental disorders are temporally correlated [41]. For instance, autism spectrum disorder (ASD) onset matches with the dendritic growth and arborisation that occurs during early childhood [42], and the expression of attention-deficit hyperactivity disorder (ADHD) symptoms at mid and late childhood and schizophrenia during late adolescence with dendritic and synaptic pruning [40,41]. ASD pathology has been associated with disrupted excitatory/inhibitory balance and abnormal connectivity of higher-order association areas [43]. Since ASD is often accompanied by an increased brain size during the first 3 years of life, it has been postulated that it could be due to a dendritic overgrowth or deficiency in pruning and maintenance of normal cell numbers [42]. ADHD and Tourette’s syndrome appear late in childhood and are characterised by a deficient connectivity in neural circuits associated with self-regulation and inhibitory capacity. Patients with ADHD have also been shown to reach peak cortical thickness later than their neurotypical counterparts [44]. The typical onset of schizophrenia occurs during adolescence or young adulthood. In schizophrenic patients, during puberty, cortical thinning occurs at a faster pace and extends to neighbouring regions compared to age matched controls [45]. The reason for the excessive thinning has been proposed to be either due to reduced dendritic branching and decreased cell number, or more commonly due to excessive synaptic pruning or irregular synaptic remodelling [46,47]. From this information, we can conclude that a failure to maintain correct dendritic maturation leads to abnormal neuronal function and circuit establishment, which ultimately results in the development of atypical behavioural symptoms associated with neurodevelopmental disorders. In this context, it is important to now consider the specific timing of dendritic maturational events and the signalling mechanisms that underpin them.

## 4. Developmental Timeline and Regulation of Neuronal Arborisation and Synapse Formation

**Figure 2:**
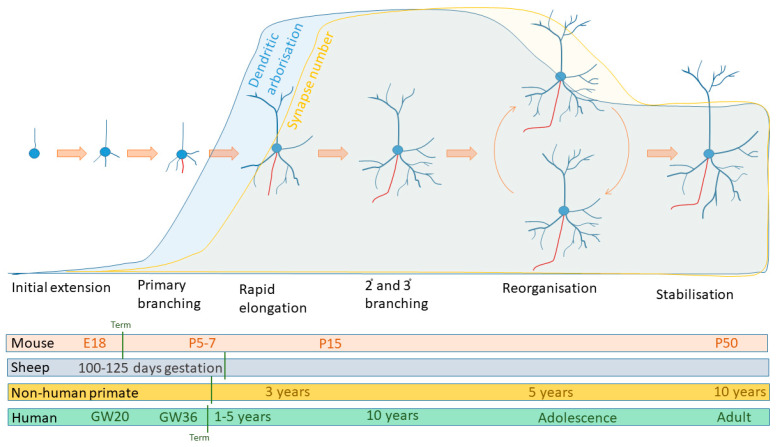
Schematic diagram summarising the comparative timeframe in which key events in dendritic arborisation occur. Dendritic branching commences with primary branch formation, immediately following neuronal migration to its final position. Processes of branching then occur to form secondary and tertiary branches, as well as branch elongation. During this period, is the initial formation of dendritic spines and, subsequently, of synapses. Reorganisation and stabilisation of the dendritic branches, spines, and synapses occur relatively late in the developmental processes. The comparative time frame of these events is shown for the mouse, sheep, non-human primate, and human brain. E—embryonic day, GW—gestational week, P—postnatal day.

The morphology of mature neurons is characterised by the multitude of highly branched processes that extend from the cell body. These neurites initially extend in a similar manner, prior to specialisation, into axons and dendrites, with spine formation part of the late dendritic specialisation in spiny neurons [2,3,8]. Phases of dendritic arborisation can be summarised as (i) growth (characterised by an initial slow phase and the subsequent fast elongation), followed by (ii) dynamic extension and retraction, leading into a final period of (iii) dendrite stabilisation (Figure 2) [2]. In addition to these neuronal-dependent processes, there is a prolonged period of pruning that is particularly dependent on environmental cues (reviewed in [1]). Therefore, there is a stereotypic element of dendritic arborisation, though there is variation in the timing of events between individual cell types, brain regions, and species.

### 4.1. Progression and Timing of Dendrite Development

Data collected from numerous species shows a general pattern of dendritic expansion during the early years of life, where the lengthening of dendritic branches and increasing branch complexity correlate with synaptic formation. This process stabilises before a period of reorganisation and synaptic pruning during early adolescence as adult patterns of arborisation and synaptic connectivity are established (see Figure 2). Typical early branching patterns, visualised with Golgi staining, show a single apical primary dendrite together with 3–9 basal dendrites [48]. These extend following the completion of migration, with the leading migratory edge thought to transition from the primary apical neurite at this point. The switch from migration to dendritic extension appears, at least in the mouse, to be facilitated by the removal of Sox11 inhibition in the early postnatal cortex [49]. The axon generally specialises from a basal neurite, while the rest contribute to the dendritic tree. In the human brain, the first dendritic branching has been reported between 16–26 weeks of gestation, increasing up to 36 weeks [50,51], and an established, though rudimentary, dendritic structure is present at term, with cortical neurons 30–55% of their maximum length [52]. A similarly established arborisation has been described at term for non-human primates [53]. In cortical neurons, the basal dendrites appear to establish their complexity earlier than the apical dendrites, with no new branch orders identified in basal dendrites after term [52]. In the mouse brain, these steps largely occur in the first postnatal week, with branching broadly equivalent to the human term, around postnatal day (P)7-10 (see comparative data in [49,54,55,56] as examples). In the sheep, another common animal for modelling developmental brain injury, dendritic arborisation within the cortex commences at around 0.7–0.85 of gestation (see data in [21,23,24]).

An analysis of layer V neurons in the human prefrontal cortex suggests that there is a rapid phase of dendritic expansion and branching that continues until 5 years of age [57]. This is then followed by a long period of local dynamic reorganisation of the dendritic branches. The majority of data regarding this period of dynamic reorganisation comes from rodent or cell culture studies exploring the molecular mechanisms regulating these stages of dendritic arborisation (discussed below). A substantial body of work in zebrafish also exists, utilising the capacity for genetically enhanced time-lapse imaging to unpick specific developmental events. While this review focuses primarily on findings from the mammalian brain, the zebrafish data is an important addition to the field and is reviewed in [58]. Data from the mouse brain clearly shows formation of primary branches by P10, with continued elaboration of secondary and tertiary branches until approximately P40 [50]. Variation in dendritic arborisation between cortical regions is detectable in the neonatal human brain, with the primary motor cortex appearing to develop first (based on identification of longer dendrites and greater number of dendritic spines) [59]. Synaptic density in the visual and auditory regions also appear to develop relatively early [60]. In both measures, the prefrontal cortex appears to lag in its maturation, with less complex dendritic branches [52] and a reduced synapse number in early life [60]. Data from the chimp brain shows a similar pattern of maturation: neurons in the prefrontal cortex continue to be less elaborate until after adolescent pruning, though they ultimately show more complexity in their branching pattern than neurons in other cortical regions [53].

Evidence for sex differences in dendrite arborisation is beginning to grow, from a mixture of in vivo and in vitro studies. These studies show a clearly increased complexity within the dendritic arbours of hippocampal neurons in male mice at P28, compared to their female counterparts [61]. This result was replicated in primary neuronal–glial cultures from P0 hippocampal tissue in the same mouse strain, and appeared to be at least partly oestrogen-dependent [61]. These differences in the formation of dendritic arbours between males and females may help explain the well-recognised sex differences in the presentation and diagnosis of neurodevelopmental disorders. While the study of Keil et al. (2017) links these sex differences to activation of the oestrogen receptor [61], a study by Beyer and Karolczah (2000) on primary mouse midbrain dopaminergic neuronal cultures suggests that oestrogen may also stimulate the growth of neurons independently of the oestrogen receptor, instead being dependent on cAMP- and PKA-derived phosphorylation of CREB [62]. Additionally, there is some evidence from mouse studies to suggest that sex differences in microglial development (innate and following inflammation) may contribute to the observed differences in neuronal arborisation and synapse number [63].

### 4.2. Dendritic Spines and Synaptic Development

Dendritic spines are microscopic membrane protrusions comprising the receptive postsynaptic compartment of synapses in the brain [15]. Spines contain neurotransmitters, neuropeptides, receptors, signalling molecules, ion channels, and other proteins that participate in synaptic transmission. Newly formed dendrites lack synapses and spines. During spinogenesis, thin finger-like dynamic protrusions named filopodia emerge from the dendritic arbour. These filopodia can form immature synapses at contacts with axons; synapses can occur along the whole length of the filopodium and at its base, and can receive multiple synapses [64]. As spinogenesis proceeds, filopodia length and frequency decreases, and dendrites start to produce thin, stubby, and mature mushroom shaped spines from retracted filopodia [65].

Dendritic spine formation on spiny neurons follows dendritic branching after a natural delay, with immature spines detectable on neurons in the human hippocampus by 36 weeks of gestation, a time when multiple dendritic branches are present [50]. Spine formation is likely to occur even earlier in the cortex, as synapses can be detected from as early as 27 weeks post-conception age, ranging from 3–10 synapse/100 μm depending on cortical region [60]. The synaptic density increases to a peak (~60 synapses/100 μm) at around 4 years of age and then declines during adolescence to an adult density of approximately 35 synapse/100 μm [60]. In the mouse cortex, spines are clearly present in immature states prior to P10 and visibly mature by P20 [54]. Synapses are detectable from P5, increasing rapidly to a largely stable number between P10–17 [66]. Patterns of synapse formation vary throughout the brain, beginning earlier in inner cortical layers (V, VI) compared to outer ones (II, III), following the inside-out development of the cortex [60].

### 4.3. Regulatory Mechanisms of Dendrite Arborisatin and Spine Formation

Actin and microtubule bundling and reorganisation are the backbone of dendritic arborisation, supporting plasma membrane expansion. Actin is located in the edges of dendrites and drives exploratory activity. Microtubules form the centre of the dendritic shaft and consolidate this newly formed shaft. The dynamic process of extension, branching, retraction, as well as the orientation of branches are coordinated by numerous signalling mechanisms. These signalling mechanisms have been described in detail by other authors (see reviews from [1,2,3]) and will be summarised here and in Table 1.

Actin monomer polymerisation allows the formation of actin filaments, stabilised by microtubules, that support axonal and dendritic branch expansion from initial actin patches (reviewed by [1]). Actin and microtubule interactions themselves are stabilised by cross linking proteins such as microtubule-actin cross-linking factor (MACF1; [94]). While microtubule invasion of filipodia supports branch formation, the disassembly of microtubules, regulated by ubiquitin protein ligase 3a (Ube3a, also called E6AP ubiquitin protein ligase), equally contributes to the retraction of dendritic branches [95]. The dynamic extension and reorganisation of dendritic branches is a highly activity-dependent process; therefore, this can be inhibited by processes that affect ATP production. As one example, the disruption of Drp-dependent fission will inhibit the production of mitochondria small enough to move into dendritic branches, and has been found to result in the substantial failure of primary branching within mouse Purkinje cells within the cerebellum (in vivo and in vitro) [96]. Alterations in microtubule transport systems can also affect branching by altering the capacity for normal actin and microtubule organisation (e.g., [97]). Relatedly, there is a substantial dependence on protein translation; therefore, alterations in mRNA transport and local protein translation also impacts neuronal arborisation (reviewed by [98]). Of note, the gene fragile X mental retardation 1 (Fmr1, the gene primarily disrupted in fragile X syndrome) is an important regulator of local mRNA translation in dendrites (reviewed by [99]).

Extrinsic regulators of arborisation included contact-dependent signalling (cell-to-cell or with the extracellular matrix), secreted chemoattractant or chemorepellent molecules, and growth factors. Protocadherins are a large family of molecules, though, to be involved in contact-dependent regulation of dendritic arborisation (and axonal extension). Clustered protocadherins have specifically been found to regulate arborisation via signalling through Rho GTPases (reviewed by [100]). A conditional knockout of γ-protocadherins in the mouse brain results in disrupted dendritic complexity, though the effect of this is primarily relatively late in cortical development, from P18 to P28 [67]. This relatively delayed age-dependent effect is particularly interesting as protocadherins have also been shown to regulate dendrite self-avoidance within arbours, a phenomenon that is clearly delineated by P12 [69]. Other proteins regulating cell-to-cell contacts that affect dendritic arborisation include dystroglycan, contactin 4 (CNTN4), and neurexin (NRXN)–neuroligin (NRLG) interactions. Dystroglycan, an extracellular matrix protein, has been shown to stimulate the growth of dendritic arbours in mouse hippocampal neurons in vitro in a manner dependent on cell division control protein 42 (Cdc42) GTPase [70]. CNTN4, a protein associated with schizophrenia and most commonly found to alter synaptic function, has also been shown to regulate arborisation of hippocampal neurons in the mouse [101]. However, in this case, the effect was relatively small, possibly reflecting the primary role of CNTN4 in synapse organisation and, therefore, the late stage at which it acts in development. Neurexin and neuroligin are adhesion proteins, which are also primarily associated with synaptic formation due to their respective presence on axon terminals and dendritic spines. It has been shown in the mouse brain that interactions between these molecules are important for the initial transient stabilisation of synapses that require activity to stabilise permanently [102]. Blocking these interactions results in reduced growth of the dendritic arbour as well as less synapse formation [102].

Secreted chemoattractants that regulate dendritic arborisation include semaphorin 3A (Sema3A), Slit guidance ligand 1 (Slit1) and ephrin A7 receptor (EphA7). Sema3A, which is a chemorepellent for axons, acts as a chemoattractant for dendrites, supporting growth and orientation of the primary apical dendrite [74] and is necessary for secondary and tertiary branching [75]. Slit1 is another secreted factor, in this case working through the Robo receptor that stimulates dendritic growth and branching [71]. Interestingly, EphA7 appears to be another regulatory molecule that has different effects throughout development, as EphA7 signalling restricts dendritic growth and early spine formation during early development (prior to P10 in the mouse) but promotes dendritic spine formation at later developmental stages [54].

The family of neurotrophic factors, including nerve growth factor (NGF), brain-derived neurotropic factor (BDNF), and neurotrophin-3 and -4 (NT-3, NT-4), that have multiple roles in regulating brain development and function, has also been identified as contributing to neuronal arborisation and synapse formation. The functions of BDNF in this context have been most widely explored and found to promote dendritic arborisation, primarily via activation of the tropomyosin receptor kinase (Trk) B receptor (TrkB, reviewed in detail by [56]). NT-3, acting through the TrkC receptor, has also been found to promote arbour growth [81]. Mature BDNF and NT-3 both have developmental-specific expression and appear to contribute primarily to the later stages of dendritic expansion. In an elegant study, Joo et al. (2014) showed that decreasing NT-3 signalling, derived from pre-synaptic neurons in an activity-dependent manner, reduced elongation of dendrites from Purkinje neurons to the pial surface of the mouse brain between P7 and P14 (resulting in disruption that continues long-term) [81]. However, other researchers have shown the capacity for inhibition of NT-3 from E21 to P7 in the mouse to result in cortical neurons with reduced apical dendrites at P7 [56], suggesting the age-specific effects may be different depending on the brain region or NT-3 source. The actions of BDNF on dendritic development are harder to summarise and can be both constitutive and activity-dependent [79]. It is likely that a molecule as pleiotropic as BDNF has substantial compartmentalisation of its signalling and signalling pathways. In support of this idea, it has recently been shown, using compartmentalised cultures of primary rodent neurons, that BDNF from post-synaptic targets is able to bind to TrkB receptors on axons and simulate dendritic arborisation via a distinct and complex intracellular signalling pathway [78]. Hepatocyte growth factor (HGF) also acts through its tyrosine kinase receptor (Met in this case), is important in stimulating many developmental events, and has been found to promote dendritic growth and branching [78,84]. Hepatocyte growth factor (HGF) also acts through its tyrosine kinase receptor (Met in this case), is important stimulating many developmental events, and has been found to promote dendritic growth and branching [84]. This is of particular interest, as HGF, as with many other growth factors, has been found to be reduced in ASD patients [86]. Similarly, neuregulins, members of the epidermal growth factor (EGF) family, have been linked to altered synaptic function in neurological disease [86]. As one example, neuregulin 1 binding to the EGF receptor ErbB4 has been shown to alter dendrite elaboration and synapse formation in glutamatergic and some GABAergic primary hippocampal neurons [103].

While the downstream signalling pathways have not been established for all extrinsic regulators of arborisation, there are clearly several common and interacting pathways that ultimately regulate actin and microtubule organisation. BDNF, for instance, is well established to alter dendritic arborisation via PI3K/Akt, cAMP response-binding protein (CREB), and other early response genes such as Arc and Rho-GTPases (reviewed in [79]). Ras- and Rho-GTPases, together with many protein kinases, e.g., glycogen synthase kinase-3 (GSK-3) and PI3K and CREB/CREB-binding protein (CBP) can be considered master regulators of dendritic arborisation (reviewed in [2]). These molecules have numerous interactions and can directly or indirectly affect cytoskeletal rearrangement. For instance, active GSK-3 phosphorylates MACF1 altering its interactions with microtublins and f-actin in the leading edges of extended neurites [94]. Knockout experiments in the mouse suggest that MACF1 is important throughout the developmental process, with knockout resulting in increased numbers of primary dendrites, but a reduced dendritic length and abnormal dendritic orientation when modified at different stages of development in vitro, as well as in vivo [94]. An alternative method of modifying these pathways, and, therefore, dendritic arborisation, is via transcriptional regulators and chromatin remodelling proteins. ARD1B is an example of a chromatin regulation molecule that affects the production of cfos and Arc (via the phosphorylation of CREB), and many downstream pathways which contribute to dendritic branching [55]. The effects of altering ARD1B binding in the mouse brain (reduced number and length of apical and basal dendrites, substantial disruption in pial contact, reduced number and increased immaturity of dendritic spines) are complex, reflecting the combined actions of these pathways [55].

While many of the mechanisms described above are activity-independent, a large part of the dynamic phase of dendritic reorganisation is activity-dependent. Signalling following neurotransmitter release and binding is essential for stabilising dendritic growth and spine and synapse formation, with many of the signalling molecules and downstream pathways described above important for the cytoskeletal reorganisation entailed (reviewed in [2]). Sin et al. (2002) showed in the optic tectum of Xenopus tadpoles that light stimulation increased dendritic arborisation by a mechanism that involved glutamate receptor signalling, resulting in reduced RhoA activity and increased Rac and Cdc42 signalling [89]. Interestingly, a subsequent study on the mouse, using dark rearing to explore activity-dependent effects on dendritic arborisation, showed similar changes but of a greatly reduced magnitude [89,104]. This suggests there could be species or brain region differences in the contribution of activity-dependent control or arborisation. Disruption in the process of neurotransmitter signalling is, therefore, clearly a contributor to neurological disorder. Transfection of an ASD-specific mutation of GRIN2 (encoding the GluN2B NMDA receptor subunit) into a subpopulation of cultured neurons in the presence of wildtype GluN2B shows that even small changes in the presence of key genes/proteins can affect dendritic arborisation [104]. In this example, there was no significant change in primary neurons, but a substantial decrease in branching, and a decrease in the length of branches at all levels of the dendritic tree [104].

To date, research on glial involvement in the successful wiring of the brain has focused on effects of astrocytes and microglia at the synapse level, specifically regulating dendritic spine number and structure and synapse formation, plasticity, and function. There is substantial evidence that astrocytes affect synapse formation through a mixture of contact-dependent mechanisms and secreted factors, including thrombospondin, cholesterol and ApoE, hevin, transforming growth factor (TFG)-β, and chondroitin sulphate proteoglycan (reviewed by [105,106]). Astrocyte–neuronal contact, regulated by γ-protocadherin, appears to be important for facilitating early phases of synapse formation [68]. Secreted BDNF appears to be a major driver of microglial-influenced dendritic spine formation, and there is also substantial evidence of microglial phagocytosis of pre- and post-synaptic elements as part of the normal pruning of excess synaptic connections, particularly those with low activity (reviewed by [107]). Phagocytosis is regulated by classical immune signalling systems, such as chemokines (specifically Cx3cr1) and complement (including C3 and C1q; [107,108,109,110]). Importantly, though beyond the scope of this review, there is also substantial data supporting a role of microglia and astrocytes in the regulation of synaptic plasticity in the adult brain (reviewed by [106,107]). Limited data is available as to whether these glial cells contribute to earlier stages of neuronal development such as dendritic arborisation, though Yang et al. (2012) [82] have shown astrocyte-dependent dendritic arborisation in the mouse brain. Interestingly, this study suggested that astrocyte knockout of Fmr1 was sufficient to induce the reduced dendritic arborisation, as a result of an over-production of NT-3 [82]. Recent data also suggests that astrocytes may regulate neuronal arborisation through the lipoprotein receptor class A repeat domain of low-density lipoprotein receptor related protein 4 (LRP4, [111]).

## 5. Potential Protective Regulatory Mechanisms and Pharmacotherapies

### 5.1. Genetic Risk Factors That Alter Neuronal Arborisation and Associate with Neurodevelopmental Disorders

The high frequency of dendrite and spine abnormalities in neurodevelopmental disorders are due to a mixture of genetic susceptibility and an altered environment during development (possibly reflecting a specific injury in some cases). Many of the genetic risk factors associated with neurodevelopmental disorders (and neurodegeneration) interact with the molecular regulators of dendritic development outline above and are summarised in Table 2. The clearest association between genetics and neurodevelopmental disorders occurs with chromosomal deletions/duplication (e.g., Methyl CpG binding protein 2, MeCP2; 22q13 etc.); however, as so many genes are affected, the neurobiology can be difficult to unpick (reviewed in [112]). MeCP2 is a transcriptional regulator that has significant effects of brain development. The effects of this gene are dose- and sex-dependent: Rett syndrome occurs in females with a reduction in MeCP2 [112,113,114], and duplication of the gene results in an increased risk of ASD and intellectual disability, primarily in males (reviewed in [113]). In terms of arborisation, deficiency in MeCP2 results in reduced dendritic branches, spine density, and abnormal spine morphology [113]. Interestingly, the pro-inflammatory cytokine interleukin-1β has been shown to interact with MeCP2 and affect its functioning (reviewed by [115]), a possible mechanism by which genetic and environmental factors could interact to increase the risk of neurodevelopmental disorder.

Some genes on these chromosomes (e.g., SH3 and multiple ankyrin repeat domain protein 2, Shank2) have independently been associated with an increased risk of neurodevelopmental disorders in the human population or experimental models, as have a number of other genetic risk factors (reviewed in [113]). Many genes identified by their association with neurodevelopmental disorders have been found to regulate synapse number and function, though recent evidence suggests that some of these may also affect dendritic arborisation under certain circumstances. For instance, the transcription factor myocyte enhancer factor 2c (MEF2c), which is associated with fragile X syndrome, has been clearly identified as a negative regulator of synapse number in the mouse brain [160]. There is recent evidence from Kamath & Chen (2020) that this transcription factor is also active in mouse cerebellar Purkinje neurons during postnatal development, and negatively regulates dendritic growth in these neurons with only minor effects on spine number [161]. Similarly, an elegant study of disease-specific mutations of Shank2 in neuronally differentiated induced pluripotent stem cells (iPSCs) has recently shown alterations in dendritic morphology in addition to synaptic changes [128]. In this study, the Shank2 mutation resulted in an overgrowth of dendritic arbours that appeared to be due to a cell autonomous sensitivity to pro-growth stimuli. There were also increases in synapse number and substantial increases in the frequency of spontaneous excitatory post-synaptic potential (EPSP) [128]. Genetic risk factors for neurodevelopmental disease effectively interact with all aspects of the molecular regulation of dendritic arborisation, and include genes for microtubule-associated and cross-linking proteins, cell adhesion and scaffolding proteins, growth factors and their receptor tyrosine-kinases, transcriptional regulators, kinases, phosphatases, and ligases (see Table 2). The genetic risk factor for neurodevelopmental disorder appears to particularly affect activity-dependent elements of arborisation and synapse formation (reviewed in detail in [113,162]). Specific mutations associated with neurodevelopmental disorders include GluN2B, GABARA3 and GABARB3, among others [113,163].

As mentioned above, the morphological changes produced as a result of a Shank2 mutation resulted in clear alterations in the electrical activity of the affected cells. By comparison, the substantial decrease in arbour complexity and length and spine formation found following knockdown of ARDIB in mice resulted in very limited alterations in the electrophysiological characteristics of the cells (no change in amplitude, frequency and decay currents of EPSPs or IPSPs) [55]. However, significant changes in the inter-event interval [55] in these cells suggests that these morphological changes may result in altered circuit function. This raises interesting questions about the links between structure and function, and clearly, more work is needed to fully understand the relationship between structural changes and function at the cell, local network, and whole brain level. In the study of Viale et al. (2019) long-term behavioural changes in mice, including hyperactivity and learning abnormalities, resulted from the 30–50% reductions in arborisation produced by altered canonical Wnt signalling, but no changes were observed in the firing properties of the cells [56].

Interestingly, altered dendritic arborisation has been shown to be one of the early events leading to seizure production in a zebrafish model of Dravet syndrome [164], suggesting that genetic risk factors may directly affect arborisation ahead of wider dysmaturation or degeneration, and disease. In this example, a mutation in the zebrafish analogue of the voltage-gated sodium channel *SCN1A*, resulted in reduced dendritic arborisation within GABAergic neurons as early as 3 days post-fertilisation (dpf), prior to the start of epileptic brain activity from 4–5 dpf, and loss of GABAergic neurons around 7 dpf [164].

Dendritic spines are clearly essential in contributing to the formation of synapses and the functional output of the resulting networks. These specialisations of the dendrite experience frequent morphology changes in response to stimuli, environment, and location, which is an essential capacity for synaptic plasticity. Depending on the circumstances, these spines may become relatively stable and increase (i.e., in response to long-term potentiation, LTP) or decrease (i.e., in response to long-term depression, LTD) their number, shape and size [64,165]. The cytoskeleton of spines is made of a dense actin matrix, whereas dendrites are constructed from microtubules [166]. Alterations in the structure and function of actin (and microtubules), as well as abnormal development of types and numbers of spines in dendrites, have been associated with both neurodevelopmental disorders and neurodegenerative disorders. In autistic brains, mutation or lack of cell adhesion molecules NRXNs and NLGNs has been associated with impaired synaptic communication and spine maturation. However, their overexpression can result in an excessive production of immature filopodia-like spines [121,167]. An abnormal expression of Shank3 or cortactin genes leads to over-excitation of postsynaptic terminals and synaptic dysfunction. Shank and Homer family proteins are postsynaptic scaffolding proteins involved in the transduction of synaptic signals from mGluR and NMDA receptors and are crucial for maturation and enlargement of dendritic spines. Abnormal expression of these genes, as seen in individuals with ASD, leads to over-excitation of postsynaptic terminals and synaptic dysfunction [64]. Calcineurin (CaN) is a calcium-sensitive phosphatase that becomes activated by an increase in calcium influx into the spine such as after TBI, glutamate excitotoxicity, and ischemia/hypoxia. Activated CaN may result in the dephosphorylation and activation of the actin-depolymerising protein cofilin. Excessive cofilin activity leads to the shrinkage and instability of spines [168,169].

### 5.2. Lessons on Treatment Strategies from Animal Experiments

As described above, the processes of dendritic formation can be categorised into overlapping steps, which are regulated by a mixture of intrinsic, extrinsic, and activity-dependent processes. Many experimental studies have provided data supporting the hypothesis that specific regulation of molecular pathways can alter arborisation and, therefore, behaviour (via alterations in synapses and cell-to-cell communication). While many of these experimental conditions involve time- and cell-dependent genetic modifications that are currently therapeutically unrealistic, they do provide some important lessons for the development of future therapeutic regimens. One such lesson is the importance of targeting pathways in an age-dependent manner. There is evidence that a single signalling pathway may produce different effects of dendritic arborisation throughout the developmental process. As an example of this, a loss of function of canonical Wnt signalling, through the expression of a dominant-negative form of a Wnt effector in the mouse (dnTCF4, [56]), reduced dendritic arborisation (decreased branch length, number and number of higher order branches) as well as resulting in long-term alterations in spine density [56]. This was primarily driven by the Wnt function from E21 to P7, shown by age-specific electroporation experiments, which was sufficient to cause dendritic malformations in an activity-independent manner. By comparison, loss of function from P21–30 caused decreased spine and synapse formation that was activity-dependent [56]. Similarly, Heppt et al. (2020) [170] have shown alterations in β-catenin signalling in adult-born hippocampal neurons in the mouse can cause an initial increase in arborisation but ultimately results in arbours less elaborated than those normally found. In the example of EphA7 signalling discussed above, age-dependent actions were found to be driven by different receptor isoforms, one signalling through mammalian target of rapamycin (mTOR) to inhibit dendritic growth and the other stimulating spine formation [54,171]. Together, these studies show that the outcome for an individual will depend very much on the specific nature of altered signalling that occurs, not just in terms of which proteins are affected, but when. This has particular relevance when considering the effect of environmental influences on brain development and risk of neurodevelopmental disorders.

It is unclear how much capacity the brain has during development to compensate for early pathological changes. There is evidence of structural “normalisation” of dendritic arbours over time. As an example, knockout of neuregulin 4, one of the epithelial growth factor family involved in neuronal development and linked with diseases such as schizophrenia and depression, was found to substantially reduce dendritic elongation and branching in the mouse brain that were detectable at P10, but not in the adult cortex [172]. The mechanism of this normalisation is yet to be extensively studied, though given that it is due to a genetic mutation, it implies either an age-specificity in the action of the gene/protein in question and/or the capacity of compensation by later developmental processes. Potential compensatory mechanisms could include dynamic processes of activity-dependent arbour reorganisation. Fundamental to these is the capacity to destabilise the cytoskeleton and allow dendritic retraction, a mechanism that can contribute to neurodevelopmental disorder susceptibility. An elegant study by Khatri and colleagues (2018) [95] in mouse brains and primary neurons showed that the E6AP E3 ligase (an ASD-associated gene) can ubiquitinate XAIP, a member of the inhibitors of apoptosis (IAP) family of proteins, resulting in its degradation and, therefore, reduced inhibition of caspase production. The subsequent increase in caspase-3 was found to destabilise tubulin, leading to dendritic retraction (partially compensated for by continued growth of other dendrites). The altered function of this gene in ASD patients may contribute to disease on its own or further disruption in combination with other risk genes or environmental challenges.

The potential for compensatory repair (developmental or pharmacological) is the primary focus of injury models of neurodevelopmental disorders, such as those resulting from inflammation or hypoxia-ischemia, that generally model an early single injury to the brain. In one such model, a developmental brain injury was produced by the premature delivery of rabbit kits resulting in altered arborisation of hippocampal neurons as well as reduced numbers of dendritic spines [173]. Oestrogen supplementation was provided to a subgroup of preterm born kits, which was found to ameliorate the spine pathology on the apical dendrites, in line with the hypothesis that reduced exposure to maternal oestrogen may be the cause of this neuropathology. Treatment with the TrkB agonist 7,8-dihydroxyflavone (DHF) was also able to ameliorate this apical spine pathology and both treatments also reduce anxiety-like behaviour in the offspring [173]. Interestingly, while reduced levels of Cdc42 and Rac proteins were identified in addition to the morphological changes produced by preterm birth, these were not ameliorated with oestrogen of DFH treatment, suggesting an alternative pathway was responsible of the spine pathology in this injury model. This study supports the idea that neurological injury can be treated by pharmacological approaches that either target the cause of injury or stimulate repair by activating the molecular development of the affected brain structures. Of course, while injury-induced neurodevelopmental disorders are generally the result of a single event, they can still have a complex disease aetiology, which needs to be disentangled if the appropriate treatment is to be selected. For instance, in the case of an inflammation-induced model of brain injury in mice, a reduced spine density has been observed in the adult brain following a systemic inflammatory insult that occurred prior to spine formation [174]. In this case, the long-term disruption to the spines is likely a result of a transient disruption in the maturation of parvalbumin interneurons [174]. Therefore, therapeutic options could include short-term anti-inflammatories early in development or the delayed use of drugs to stimulate interneuron development or spine formation. The validity of this approach remains to be confirmed.

In addition to the neuronal intrinsic genetically regulated over-pruning described above, there is also the possibility of glial-induced over-pruning. This is another area of potential overlap between genetically regulated and injury-induced neurological injuries. Interestingly, microglial and astrocyte dysfunction has been associated with a wide range of neurodevelopmental disorders (reviewed by [105,106]) and mutation of disease-associated genes specifically in astrocytes is able to recapitulate many aspects of disease [82] (reviewed in [106]). Moreover, there is evidence that phagocytic activity of wild-type microglia (derived from a bone marrow transplant) can ameliorate neuronal spine morphology disruptions and behavioural deficits in a genetic mouse model of Rett’s syndrome [175]. The reliance of microglial-derived regulation of dendritic spines and synapses on classical immune signalling molecules suggests a potential mechanism by which environmental changes and injury may contribute, along with genetic factors, to neurodevelopmental disorders. Sellgren et al. (2019) showed that induced microglial cell with the C4 risk variant (from iPSC derived from schizophrenic patients) increased phagocytic engulfment of synaptic structures in neurons also derived from iPSCs. Minocycline treatment, as an example of an anti-inflammatory therapy, was shown to reduce this microglial phagocytosis of synapses in this in vitro model [176]. A retrospective study conducted by the same researchers of individuals prescribed antibiotics, including minocycline and doxycycline, for at least 90 days, showed a decreased incidence of psychosis in these individuals, compared to the population as a whole [176].

As an interesting aside, one of the early pathological signs of Alzheimer’s disease is the presence of abnormal, and loss of, spines [177]. There is the potential that this is driven from neuroinflammation relatively late in disease progression, but as subtle neuropathology continues to be identified early and prior to clinical signs, alternative options should also be considered. One of the proteins that has been seen to be most affected by Aβ-plaques is cofilin, an actin filament depolymerising protein, which is excessively activated by calcium excitotoxicity and via Rho-GTP-ase activity and PI3K/Akt/mTOR signalling pathway. This results in suppression of actin dynamics, which leads to a decrease in spine stability and shrinkage, and ultimately to spine collapse [8,178]. Similarly, Parkinson’s disease is characterized by a loss of spines in striatal neurons: for instance, medial spiny neurons experience a loss of 30–40% of spines as the disease progresses [179,180]. A candidate for spine loss in Parkinson’s disease is CaN (also known as PP2B), whose excessive activation activates the signalling pathways of MEF2 and cofilin as well as leucine-rich repeat kinase 2 (Lrrk2), which results in spine shrinkage and loss. Therefore, as we move towards identifying realistic pharmacological options for supporting dendritic arborisation and spine formation, we should keep in mind that they may have a utility outside the field of neurodevelopmental disorders.

### 5.3. Treatment Selction for Future Pharmacological Intervention

When considering potential therapeutic options for neurodevelopmental disorders, there is a common need for supporting the growth and stabilisation of dendritic arbours. The response of spines in these conditions is more complex, and it is likely that a second, more disease-specific, therapeutic option is required at a later developmental timepoint to support the appropriate development of the specialised structures. In the case of dendritic arborisation, in most situations, there appears to be sufficient growth of primary dendrites, so it is likely that treatment during intermediate stages of development to support secondary and tertiary branching and elongation may be most beneficial. To produce these effects, numerous therapeutic options might be considered. Currently, the most therapeutically realistic of these is probably the use of growth factors. These have the desired effects on arborisation and can be stably produced and delivered. As described above, the TrkB agonist DHF has been shown to support dendritic growth in an in vivo rabbit model of preterm birth. The addition of recombinant growth factors has been used successfully in vitro (e.g., [84]). In a clinical situation, it may be possible to administer these factors by mini-pump into the cerebrospinal fluid, though it may be that a more technologically advanced approach could be utilised. A multidisciplinary approach has led to the development of a biodegradable microcapsule containing NGF that can be delivered to the targeted cells. They successfully showed that NGF-loaded microcapsules increase neurite outgrowth, branching complexity, and synapse formation in primary rat hippocampal and astrocyte co-cultures [181]. This work has the potential to enable neurite morphological and functional reconstruction and possible circuit regeneration by delivering capsules directly into the affected area. Although further in vivo studies are required to confirm this, they have successfully demonstrated, in a previous publication, that targeted and in situ delivery is possible and effective; direct in vivo micro-injection of sodium-channel blocker QX-314 (to most likely inhibit TRPV1 pain receptor activation) filled micro-capsules significantly reduced pain in a persistent inflammatory pain model in rats [182]. This technology could open new avenues in the development of personalised medicine to tackle individual and unique but also common and widely shared abnormalities.

Rac, CDC42, Rho, and CREB contribute to core signalling pathways in dendritic arborisation, as described above, and may, therefore, make appealing therapeutic targets with broad applicability. The Rho-associated coiled-coil containing the protein kinase (ROCK) inhibitor fasudil has been used in a mouse model of chronic stress induced disruption to hippocampal dendritic arborisation [183]. This particular study does not have substantial applicability to the current clinical question, being used in the adult mouse as a pre-treatment. However, it does provide proof-of-principle that systemic administration of an inhibitor to this pathway can ameliorate a substantial decrease in spine number and prevent behavioural disruption [183]. A potential concern for targeting these therapeutically is that they may be too ubiquitous for the sort of selective correction of arbour disruption that is likely to be required. However, this could also be an advantage, as it could work on diseases with a different genotype but equivalent phenotypes. The study of Hayashi-Takagi et al. (2015) has shown that inhibiting Rac1 can be used effectively to improve learning and memory [184]. This study is one of a number using genetic techniques to deliver targeted therapy, an approach also used successfully by Shields (2017) to specifically reduce AMPA-receptor activation on dopaminergic neurons for treatment and to reduce Parkinson’s disease-like behaviours [185]. Currently, it is unclear that such “gene-modification-facilitated” drug delivery is realistic in a clinical population, but with either improvements in gene-targeting methods, the specific stratification of patients, or the development of other targeted-delivery systems, this sort of cell-specific therapy may be a reality in the future.

There is a long phase of activity-dependent reorganisation of the cortical networks that is generally considered to be a natural period of plasticity that allows catch-up growth or increased destabilisation and pruning of unwanted connections as required. However, such normal processes may be impaired if there is cell loss or delayed cell maturation (including arborisation) at critical periods to facilitate the appropriate activity-dependent reorganisation. While some of our therapeutic aims may be to prevent these earlier events, supporting activity-dependent processes at later developmental stages may be an alternative option. Allosteric modulation of neurotransmitter receptors may be an option for this. Certainly, negative allosteric modulation of mGluR5 has been found to ameliorate repetitive behaviours and disrupted social interaction in a genetic model of ASD [186]. A case has also been made for the modulation of mGluR2 and 3, as a result of data from a mouse model of schizophrenia-like disease following prenatal stress [187]. There is also evidence that positive or negative allosteric modulation of the mGluR family can alter glial cell function [188], which may help ameliorate disrupted development caused by the activation of these cells. Importantly, the work of LaCrosse et al. (2015) demonstrates that allosteric modulation of mGluR5 is also able to modify dendritic arborisation and spine formation in the rat brain [189]. More work would be required to ensure that a treatment of this sort was given at the correct time, and it may be that at specific stages of development, or in the context of a specific genetic backgrounds, enhanced inhibitory signalling would be more appropriate.

A final class of potential therapies that are already clinically realistic are the anti-inflammatory agents. The work of Sellgren et al. (2019) [176], described above, highlights the potential for anti-inflammatory therapy to reduce glial over-activity and excessive phagocytosis of the developing dendritic spine. Acute inflammation is commonly associated with MMP-9 activation, which can proteolytically degrade β-dystroglycan, leading to reduced neurite extension [70]. This is a potential mechanism for arborisation disruption in situations like preterm birth and hypoxic-ischemic encephalopathy, where there is a high proinflammatory burden [190,191]. There is also evidence that, with excitatory–inhibitory imbalance, a situation commonly thought to exist in most neurodevelopmental disorders, excessive excitation may in itself be pro-inflammatory [192], and there may be, therefore, a potential role for mild anti-inflammatory therapy to ameliorate this and prevent processes such as glial-induced over-pruning in neurodevelopmental disorders. There is a concern that the use of anti-inflammatory agents during early development may detrimentally affect normal developmental processes [190], but this is less relevant at the comparatively late developmental stages that would be appropriate for the treatments proposed here. It should be noted that steroidal anti-inflammatories are probably not ideal therapeutic options. Certainly, long-term elevation of corticosterone in the adult mouse has been associated with a decreased number and length of basal dendrites that persists beyond the treatment period [193].

## 6. Conclusions

In the complex neurobiology of neurodevelopmental disorders, altered dendritic arborisation and spine formation play a key role. The carefully orchestrated molecular regulation of arbour growth presents multiple potential pathways for therapeutic intervention in these conditions to normalise the developmental trajectory. Some of these targetable pathways may be sufficiently ubiquitous to be useful in a wide range of disorders, though there is a likelihood that disease- (and cell-) specific targeting will ultimately provide the most therapeutic gain. There is clear evidence from the body of scientific research reviewed here that the timing of intervention is important, but also that early treatment is not necessarily the only therapeutic option, and that gains may be achieved by a transient-targeted alteration of key process in dendritic arborisation and spine formation. For this aim is to be attained, we require a coordinated and therapeutically directed focus to research this field in the coming years.

## Figures and Tables

**Table 1:** Extrinsic regulators of dendritic arborisation and spine development.

Molecule Type	Molecule Name	Known Interacting Proteins/Pathway	Effects on Dendritic or Spine Development	Refs.
**Adhesion molecules**	Protocadherin	Rho GTPases	Promotes dendritic growth and branching	[67,68,69]
	Dystroglycan	Extracellular matrix protein, cleaved by activated MMP-9	Stimulates dendritic growth	[70]
**Chemoattractant**	Slit1	Binds to Robo receptor. Activates Slit/Robo pathway	Promotes dendritic growth and branching	[71,72]
	EphA7	Downstream of EphA receptor signalling	Activation restricts dendritic growth and early spine formation	[73]
	Sema3A	Semaphorin pathway	Stimulates dendritic growth of secondary and tertiary branches, controls branch orientation	[74,75]
**Neurotrophic factors**	NGF	Binds TrkA, can activate small GTPase Rac1 in a PI- 3kinase–dependent manner	MAP kinase and PI-3Kinase pathways that regulate Rho GTPase activity leads to actin cytoskeleton dynamics, neurite elaboration and rapid dendritic remodelling	[76]
	BDNF	Binds to TrkB or p75 receptor		[76,77,78,79,80]
	NT-4	Binds to TrkB		[76]
	NT-3	Binds to TrkC		[76,81,82]
**Hormone/growth factors**	IGF-1	Binds to IGFR1		[83]
	HGF	Binds to MET tyrosine receptor kinase and downstream C-Met signalling pathway	Stimulates dendritic growth and branching of basal and apical branches	[84,85,86]
**Notch ligands**	Delta	Binds to Notch type 1 cell-surface protein, Notch signalling	Nuclear translocation and binding to [CBF1/RBPJk/Su(H)/Lag1] family of transcription factors. Promotes contact-dependent inhibition of neurite outgrowth	[87]
	Serrate			
**Wnt ligands**	Wnt7b	Activation of non-canonical Wnt pathway via Rac and JNK activation	Increases dendrite numbers and promotes dendritic growth and branching complexity	[88]
**Small GTP-binding proteins**	Rac	Rac signalling pathway	Induces actin polymerisation, assembly of contractile actin and myosin filaments, formation of lamellipodia	[89,90,91]
	Cdc42	Cdc42 signalling pathway	Establishes correct cell polarity, actin filament assembly and formation of filopodia	
**Transcription regulators**	CREST	Activation upon Ca2+ binding. Part of CREST-CBP-BAG250/BRG-1 complex.	Regulates calcium-dependent dendritic development. Stimulates dendritic branching complexity and outgrowth of basal dendrites	[92]
	CaMKIV	Activation upon Ca2+ binding. CaMK signalling pathway. Phosphorylates CREB.	Stimulates activity-dependent dendritic growth	[93]

Abbreviations: Slit1—Slit guidance ligand 1, Epah7—ephrin receptor A7, Sema3A—semaphorin 3A, NGF—nerve growth factor, BDNF—brain-derived neurotrophic factor, NT—neurotrophin, IGF1—insulin-like growth factor 1, HGF1—hepatocyte growth factor 1, Rac—Rac family 1 small GTPase 1, Cdc42—cell division cycle 42, TrK—tyrosine kinase receptor, CREST—calcium-responsive transactivator, CaMKIV—calmodulin-dependent protein kinase IV, CREB—cAMP-response element-binding protein.

**Table 2:** Genes (and proteins) that confer risk for neurodevelopmental and degenerative disorders. Information summarises the known interaction pathways and downstream effects on dendritic arborisation and spine development.

Gene (Protein) Name	Protein Type	Functional Role/Known Interacting Proteins/Regulators	Gene Alteration Associated to Neurological Disease	Effect on Spines/Synapses in Pathology	Effect on Dendrites in Pathology	Refs
**NLGN3 (neuroligin 3)**	Cell adhesion proteins	Interacts with NRXN1, Epac and Shank family	ASD	↓ spine density, ↓ synapse stability	↑ branching complexity	[116,117,118]
**NLGN4 (neuroligin 4)**		Interacts with NRXN1, Epac and Shank family	ASD	↓ synapse and spine density, ↓ excitatory synapse number		[119,120]
**NRXN1 (neurexin 1)**		Ligand for NLGN3/4	ASD, Schizophrenia, Epilepsy	↓ spine density, ↓ synapse stability	↓ dendritic length	[121,122,123,124,125]
**SHANK2 (shank 2)**	Postsynaptic scaffolding proteins	Signals downstream of NLGN3/4	ASD, Mental retardation	abnormal spine size, ↓ spine density,	↑ branching complexity, ↑ dendrite number	[126,127,128,129]
**SHANK3 (shank 3)**			ASD, Schizophrenia	↓ synapse and spine density	↓ branching complexity	[130,131,132,133]
**RAPGEF4 (Epac)**	Rap guanine nucleotide exchange factor	Binds to NLGN3, synaptic regulatory pathway	ASD	↑ spine density and area	↓ branching complexity, ↓ dendritic number, ↑ dendrite length	[134,135]
**TSC1 (tuberous sclerosis protein 1)**	Tumour suppressor protein	Repress mTOR signalling pathway	Tuberous sclerosis complex, ASD, Epilepsy	↑ spine length, ↓ spine density		[136,137]
**TSC2 (tuberous sclerosis protein 2)**				enlarge spine heads, ↓ synapse density		[136,137]
**PTEN (phosphatase and tensin homolog)**	Tyrosine phosphatase, tumour suppressor protein		ASD, macrocephaly	↑ spine density	dendritic hypertrophy	[137,138]
**FMR1 (Fmrp translational regulator)**	Multifunctional polyribosome-associated RNA-binding protein	mRNA trafficking	Fragile X syndrome	↑ spine density, ↑ immature spine morphologies	↓ dendritic length and number	[14,139,140]
**MeCP2 (Methyl-CpG Binding Protein 2)**	Transcriptional regulator chromosomal protein	Repress transcription from methylated gene promoters	Rett syndrome	abnormal spine morphology, ↓ spine density	↓ dendritic length and number	[14,77]
**UBE3A (E3 ubiquitin ligase)**	Ubiquitin ligase	Part of the ubiquitin protein degradation system	Angelman syndrome	maternal deficiency: ↓ spine density in offspring	maternal deficiency: ↓ dendritic length in offspring	[95,141]
**ERBB4 (ErbB4, Erb-B2 Receptor Tyrosine Kinase 4)**	Postsynaptic receptor tyrosine kinase	Receptor for NRG1	Schizophrenia	GoF: ↑ spine density, area and excitatory synaptic transmission; LoF: ↓ spine density and size	GoF: ↑ branching complexity; LoF: ↓ dendritic length and number	[103,142,143]
**DISC1 (Disrupted in schizophrenia)**	Involved in scaffolding proteins in spines	Interacts with kalirin-7 via activation of Rac1	Schizophrenia	↓ spine size and density	↓ dendritic length	[144,145,146]
**DGCR8 (DGCR8 Microprocessor Complex Subunit)**	miRNA processing	Biogenesis of microRNAs	22q11.2 microdeletion syndrome (schizophrenia)	↓ spine density	↓ branching complexity	[147]
**ZDHHC8 (Zinc Finger DHHC-Type Palmitoyltransferase 8)**	Palmitoyl transferase	Palmitoylates PSD95		↓ spine size	↓ branching complexity	[148]
**KALRN (Kalirin RhoGEF Kinase)**	Kinase	Regulates effect of DISC1 on spine morphology, PAK	Schizophrenia, Alzheimer’s disease	spine loss, regulates spine morphogenesis and is upstream regulator of PAK in spines.	↓ branching complexity	[149,150]
**APOE4 (apolipoprotein E4)**	Apoprotein	Catabolism of triglyceride-rich lipoproteins	Alzheimer’s disease	↓ spine density	↓ dendritic length and branching complexity	[151,152]
**PAK (p21-activated kinase)**	Regulator of actin assembly	Kalrini-7; downstream effector of Rac	Schizophrenia, Alzheimer’s disease	GoF: abnormal spine morphology; LoF: ↓ spine density, ↓synapse stability	GoF: ↑ branching complexity and dendrite number; LoF: ↓ branching complexity and dendrite number	[153,154,155]
**CaN/PP2B (calcineurin)**	Calcium sensitive phosphatase	Interacts with GSK-3beta, MEF2, Lrrk2 and Cofilin	Alzheimer’s and Parkinson’s disease	↓ spine density, ↓ synapse stability	dendritic dystrophy	[8,156,157]
**MACF1 (Microtubule Actin Cross-Linking Factor 1)**	Crosslinking protein	Interacts with F-actin to regulate cell polarization	Parkinson’s disease	abnormal spine morphology, ↓ spine density	↓ dendritic length and branching complexity	[94,158]
**MAP2 (Microtubule Associated Protein 2)**	Microtubule-associated protein	Microtubule assembly	ASD, Schizophrenia	↓ spine density,	↓ dendritic length and number	[12]
**MARK1 (Microtubule Affinity Regulating Kinase 1)**	Serine/threonine-protein kinase	Cell polarity and microtubule dynamics regulation	ASD		↓ dendritic length and abnormal morphology	[159]

Abbreviations: ASD—Autism Spectrum Disorder, GoF—gain of function, LoF—loss of function, ID—Intellectual Disability.
